# Heart Rate Variability Changes in Patients With Major Depressive Disorder: Related to Confounding Factors, Not to Symptom Severity?

**DOI:** 10.3389/fnins.2021.675624

**Published:** 2021-07-05

**Authors:** Jan Sarlon, Angelica Staniloiu, Andreas Kordon

**Affiliations:** ^1^Center for Affective, Stress and Sleep Disorders, Psychiatric Clinics, University of Basel, Basel, Switzerland; ^2^Oberbergklinik Hornberg, Hornberg, Germany; ^3^Department of Psychology, University of Bielefeld, Bielefeld, Germany; ^4^Department of Psychology, University of Bucharest, Bucharest, Romania; ^5^Department of Psychiatry, University of Freiburg, Freiburg, Germany

**Keywords:** depression, autonomous nervous function, symptom severity, confounding factors, blood pressure, HRV

## Abstract

**Background:**

The aim of this study was to assess the electrophysiological and other influencing factors correlating with symptom severity in patients with major depressive disorder (MDD) under three different conditions: baseline, stress exposure, and relaxation following stress exposure.

**Methods:**

Symptom severity was assessed using the Beck Depression Inventory (BDI-II) in 89 inpatients (37 women; mean age 51 years) with MDD. Resting heart rate (RHR), heart rate variability (HRV), respiration rate (RR), skin conductance (SC), and skin temperature (ST) were recorded at baseline for 300 s, under stress exposure for 60 s, and under self-induced relaxation for 300 s. Age, nicotine consumption, body mass index, and blood pressure were evaluated as influencing factors.

**Results:**

The BDI-II mean score was 29.7 points. Disease severity correlated positively with SC elevation under stress exposure and with a higher RR in the relaxed state, but no association was found between HRV and symptom severity. Age and higher blood pressure were both associated with lower HRV and higher RHR.

**Conclusion:**

The results indicate that, in patients with MDD, changes in the autonomic nervous system (ANS) are complex; and the assessment of ANS reactivity to stressors is useful. Elevated blood pressure might be underdiagnosed, although it is already relevant in patients with MDD in their early 50s.

## Introduction

Major depressive disorder (MDD) has been repeatedly associated with dysregulation of the autonomic nervous system (ANS) (Schulz et al., 2010; Chang et al., 2012; Stapelberg et al., 2012; Liang et al., 2015; Alvares et al., 2016; Yeh et al., 2016; Shinba, 2017; Huang et al., 2018). Heart rate variability (HRV) has been used in the majority of studies using electrophysiological parameters to assess ANS (Kemp et al., 2010; Stapelberg et al., 2012; Liang et al., 2015; Alvares et al., 2016; Yeh et al., 2016; Huang et al., 2018); other electrophysiological parameters, such as resting heart rate (RHR), skin temperature (ST), skin conductance (SC) or its response (SCR), blood pressure (BP) variability (BPV), and baroreflex sensitivity (BRS), have also been used (Bonnet and Naveteur, 2004; Salomon et al., 2009; Lin et al., 2011; Thorell et al., 2013; Schumann et al., 2017; Tessier et al., 2017; Hu et al., 2019). In the following, some findings of the mentioned studies are summarized. Regarding HRV-measures, Alvares et al. (2016) demonstrated substantial reductions in HRV across all psychiatric disorders, Huang et al. (2018) demonstrated the bidirectional association between depression and autonomic dysregulation, and Kemp et al. (2010) demonstrated reduced HRV in MDD. There are findings showing both lower (Bonnet and Naveteur, 2004) and higher resting SC in depressed patients (Lin et al., 2011). In the same article, resting SC was higher and resting ST lower in subjects at high risk for depression, with decrease of SC and increase of ST during stress. A meta-analysis of studies investigating SC in depressed patients suggested that electrodermal hyporeactivity is sensitive and specific for suicide (Thorell et al., 2013). Higher RHR was observed in depression (Schumann et al., 2017) or could predict depression (RHR over 30 min in the morning in patients in the first week after stroke was a predictor of depression at 3-month follow-up measured by Hamilton depression scale) (Tessier et al., 2017).

Furthermore, the use of other methods to assess sympathetic nervous system activity, such as noradrenaline spillover methodology (Veith et al., 1994; Dawood et al., 2007) or direct muscle sympathetic nerve recording (Scalco et al., 2009), in patients with depression can be found in the literature.

Despite the rising popularity of HRV measurements in the assessment of stress/ANS, there are three main concerns regarding HRV studies in patients with MDD. First, there is a wide variety of HRV measurement protocols in the current research (Young and Leicht, 2011; Vila et al., 2019). Second, the varying validity and reliability measures across the different HRV parameters must be considered (Young and Leicht, 2011; Pereira et al., 2017; Chen et al., 2020). Third, most studies target primary ANS changes at baseline, not as a reaction to a stressor. Of the 273 identified studies using HRV in depression, only 26 met the inclusion and exclusion criteria for a review of HRV reactivity to stressor tasks in relation to depression (Hamilton and Alloy, 2016).

Furthermore, the current research suggests dysregulation in terms of abnormal autonomic responses to physical (cognitive, social, or emotional) stressors in patients with MDD compared with those in healthy controls (Rottenberg et al., 2007; Liang et al., 2015; Hamilton and Alloy, 2016), with autonomic shifts to sympathetic dominance at rest, but toward parasympathetic dominance in response to stress (Liang et al., 2015; Shinba, 2017).

Concerning symptom severity and ANS function in patients with MDD, previous research is characterized by heterogeneity of results. While some studies have demonstrated the impact of disease severity on ANS dysfunction (Kemp et al., 2010; Lesnewich et al., 2019; Giurgi-Oncu et al., 2020), others have contrary findings (Koschke et al., 2009; Koenig et al., 2016). In particular, the use of antidepressant medication may moderate the relationship between depression severity and cardiovascular function (Lesnewich et al., 2019; Giurgi-Oncu et al., 2020). Some authors consider antidepressants to play an important role in ANS changes in patients with MDD (Dawood et al., 2007; Licht et al., 2008).

These diverse results can be explained by methodological shortcomings in the application of HRV assessment, exploring ANS functions, particularly by HRV, or by a narrow interpretation of this variable. The critical points mentioned in the literature include among others the incorrect use of low frequency/high frequency (LF/HF) as a marker of “sympatho-vagal balance” (Billman, 2013; Reyes del Paso et al., 2013), the confounding effects of respiration (Widjaja et al., 2013; Beda et al., 2014), and the weak correlations within HRV parameters and other electrophysiological parameters (Thomas et al., 2019) or the problematic robustness to artifacts in real-world recordings (von Rosenberg et al., 2017). Thus, a broader analysis using more electrophysiological parameters at the same time seems to be important to clarify ANS function (Schumann et al., 2017; Sarlon et al., 2018).

## Aims and Hypotheses

A previous pilot study (unpublished data) of 32 patients with MDD and 32 healthy controls showed significantly higher RHR and respiration rate (RR) and significantly lower HRV parameters in patients with MDD than in healthy controls at baseline. The disease severity assessed using the Beck Depression Inventory (BDI-II) had no impact on the measured parameters. For that reason, we aimed to confirm the results on a larger sample size of depressed patients only with the same electrophysiological battery; however, we conducted the test under three different conditions: baseline, stress exposure, and relaxed state following stress exposure.

This is of interest, as the findings of electrophysiological changes and symptom severity are inconsistent, and the use of a wider range of electrophysiological parameters under different conditions might help clarify the function of the autonomous nervous system in patients with MDD.

We expected a negative correlation between SC response under the stress condition and symptom severity.

A further aim of this study was to investigate the role of influencing factors, such as age, sex, and BP, on the different testing conditions.

## Materials and Methods

### Participants

Ninety-eight inpatients were included in this study. The inclusion criteria for the MDD group were a diagnosis of MDD based on structural interviews and the International Classification of Diseases (ICD-10, 10th edition), as well as alcohol abstinence for 4 weeks. Only unipolar depression (first episode or recurrent) in the acute phase was considered; patients with bipolar affective disorder were excluded (one subject). Further exclusion criteria were diabetes mellitus (one subject), other manifest forms of psychiatric comorbidity (three subjects), manifest neurological disorder (one subject), and non-compensated hypertonia or heart disease, cancer, or other serious medical conditions. Patients with a pathological thyroid-stimulating hormone (THS) score (two subjects) or an elevated leukocyte count (one subject) were also excluded.

The final sample consisted of 89 inpatients (37 women, 42%; mean age, 51 years; SD, 11.28; range, 19–68) with a diagnosis of MDD without any relevant comorbidities. Thirty-one patients were drug naïve, and 58 were on psychiatric medication. The most frequent antidepressants taken were selective serotonin reuptake inhibitors (SSRIs) (escitalopram 12, sertraline seven, and citalopram three), followed by venlafaxine (13), mirtazapine (13), bupropion (six), vortioxetine (two), and tianeptine (two).

Other medications consisted of antihypertensive drugs (candesartan in three cases, telmisartan and ramipril in two cases each, amlodipine and torsemide in one case each), levothyroxine (six patients), proton pump inhibitors (pantoprazole in two and omeprazole in one case), acetylsalicylic acid (two cases), diclofenac (one case), and asthma spray (two patients).

A comorbidity of arterial hypertension was observed in 15 patients, nine of whom were on medication. Previous or current hypothyroidism was observed in seven patients (six on medication). Minor injuries in the past had occurred in three participants, and six participants had allergies or asthma.

All participants were introduced to the experimental protocols and provided written consent prior to participation. The experimental protocol was approved by the Ethics Committee of the institute (Psychiatric Hospital, Oberbergkliniken, Hornberg, Germany, Nr. 2018-046) where the data were collected.

To assess the severity of depressive symptoms, the German version of the BDI-II (Kühner et al., 2007) was administered prior to the electrophysiological measurements. Body mass index (BMI), average nicotine consumption, and pack-years of smoking were assessed.

### Measurement Protocol

Electrophysiological data were acquired using the NeXus-10 system (NeXus-10 Mark II^®^, BioTrace+, Mind Media B.V., Herten, Netherlands) and were recorded at a sampling rate of 1,024 Hz.

For heart rate and HRV recording, a blood volume pulse finger clip sensor was used. To measure SC, Velcro tape with integrated Ag/AgCl electrodes was placed at the middle phalanx of the index finger and the ring finger of the left hand. For the measurement of ST, the NeXus temperature sensor was placed on the middle finger of the left hand and taped to the finger at two positions to ensure stability.

To measure RR, an elastic belt with a breathing sensor was fixed around the lower thorax at the diaphragm, according to the manufacturer’s instructions. The same procedures were used for all study participants to ensure that the data from all individuals were comparable. All measurements were taken in awake subjects in the sitting (slightly inclined) position and in the same room. To reduce the impact of pretest movements, all subjects were asked to breathe normally and not move for 5 min prior to placement of the electrodes.

For baseline, the subjects stayed in this sitting, slightly inclined position for 5 min with their eyes open and breathing normally, and were required not to talk and to avoid all unnecessary movements.

Among emotion-induced stressors, various recall tasks designed to elicit specific emotions have been used (Cyranowski et al., 2011). In our study, we decided to use a recall of unpleasant stressful experiences of a medium intensity as a stress exposure. We asked participants to imagine an unpleasant stressful situation from the past of medium intensity and to mark the stress intensity on a visual analog scale (VAS; a horizontal line, 100 mm in length, anchored by word descriptors at each end, and numbered from 0 to 10). The VAS score was determined by measuring in cm from the left end of the line to the patient’s mark. The mean stress intensity measured by VAS was 4.85 (SD = 0.67).

A relaxed state followed the stress exposure: patients were instructed to try to relax as much as possible, with their eyes either closed or open, staying in the same position, and avoiding all unnecessary movements.

### Preprocessing and Analysis

Recording and primary analysis of all electrophysiological parameters were performed using the BioTrace+ software. For the analysis of HRV, time- and frequency-domain parameters and records 5 min in length with a sampling rate of 1,024 Hz were used, according to recognized standards (Malik et al., 1996). The power frequency spectrum of HRV was subsequently quantified in standard frequency-domain measurements, including total variance, HF (0.15–0.4 Hz), LF (0.04–0.15 Hz), very low frequency (VLF; 0–0.04 Hz), and HF/LF ratio. For the time domain, principal parameters such as beat-to-beat interval (RR or NN-interval), square root of the beat-to-beat interval (SDNN), and root mean square of successive differences (RMSSD) were used.

Prior to the analyses, all parameters were visually controlled on a 15-s window, and artifacts in the RHR and HRV parameters due to movement were removed. Artifacts due to device failure in the SC or ST were also removed. Measurements with more than 5% artifacts were excluded; this was the case in four measurements of SC as well as in five measurements of RHR and HRV parameters.

Statistical analyses were performed using R-Studio software (version 1.2). The normality of the distribution was assessed using the Shapiro test. To assess differences between groups, a Wilcoxon signed-rank test for non-normally distributed data was used. For normally distributed variables, a Pearson correlation analysis was used; non-normally distributed variables were assessed for correlation using Spearman’s correlation.

## Results

The mean BDI-II score was 29.66, which corresponds to a severe depressive episode as a major diagnosis. Twenty-one subjects (23.6%) were smokers, the mean tobacco consumption was 4 pack-years (SD = 9.88), the mean BMI was 25.5 (see also Table 1 summarizing the study population), and the mean BP was 138/86 mmHg.

**TABLE 1:** Basic characteristics of the study population.

	Age	BMI	BDI	Sex
Mean	50.61	25.52	29.66	F = 37
Minimum	19	18.00	11	M = 52
Maximum	69	34.60	60	
SD	10.34	4.02	10.71	

*BDI, Beck Depression Inventory; BMI, body mass index; SD, standard deviation.*

There were no between-group differences in SC and ST. A significantly elevated RHR was observed under stress exposure (*M* = 79.22) compared with the baseline (*M* = 74.11, *p* = 0.0018) or relaxed state (*M* = 73.80, *p* = 0.0011). The RR under stress was also significantly higher than that at baseline (*M* = 17.05 vs. 15.49, *p* = 0.015) or in the relaxed state (13.21, *p* = 1.0 × 10^–8^). The RR in the relaxed state was significantly lower than that at baseline (*p* = 0.36 × 10^–4^). Regarding HRV parameters, SDNN was significantly higher in the relaxed state than under stress exposure (38.18 vs. 30.60, *p* = 0.019); VLF under stress exposure was significantly lower than that at baseline (45.95 vs. 543.78, *p* = 2.2 × 10^–16^) or in the relaxed state (384.76, *p* = 2.2 × 10^–16^); the same constellation could be observed for LF (72.90 stress exposure vs. 1895.97 baseline, *p* = 2.2 × 10^–16^; as well as vs. 2,087.26 relaxation, *p* = 2.2 × 10^–16^) and HF (stress mean: 38.22 vs. baseline mean: 624.32, *p* = 2.2 × 10^–16^ and vs. relaxation mean: 602.95, *p* = 2.2 × 10^–16^).

Table 2 presents all the results of the electrophysiological parameters across the three different conditions (baseline, stress exposure, and relaxation).

**TABLE 2:** Electrophysiological parameters across the three different conditions (baseline, stress exposure, and relaxation).

Mean ± SD	SC	ST	RHR	RR	SDNN	RMSSD	VLF	LF	HF	LF/HF
Baseline	1.51 ± 1.28	32.54 ± 3.56	74.11 ± 10.62	15.49 ± 4.15	35.58 ± 19.99	25.07 ± 14.68	543.78 ± 1053	1894.97 ± 4052	624.32 ± 892	3.22 ± 3.23
Stress exposure	1.79 ± 1.63	32.26 ± 4.16	79.22 ± 10.25	17.05 ± 4.36	30.60 ± 17.00	21.43 ± 10.77	45.95 ± 75.47	72.90 ± 143	38.22 ± 101	2.94 ± 3.61
Relaxed state	1.64 ± 1.50	33.14 ± 3.34	73.80 ± 10.71	13.21 ± 4.11	38.18 ± 22.06	26.19 ± 18.59	384.76 ± 460	2087.26 ± 3393	602.95 ± 905	4.49 ± 6.02

*HF, high frequency; HRV, heart rate variability; LF, low frequency; RHR, resting heart rate; RMSSD, root mean square of successive differences; RR, respiration rate; SC, skin conductance; SDDN, square root of the beat-to-beat interval; SD, standard deviation; ST, skin temperature; VLF, very low frequency.*

### Symptom Severity and Medication

**FIGURE 1:**
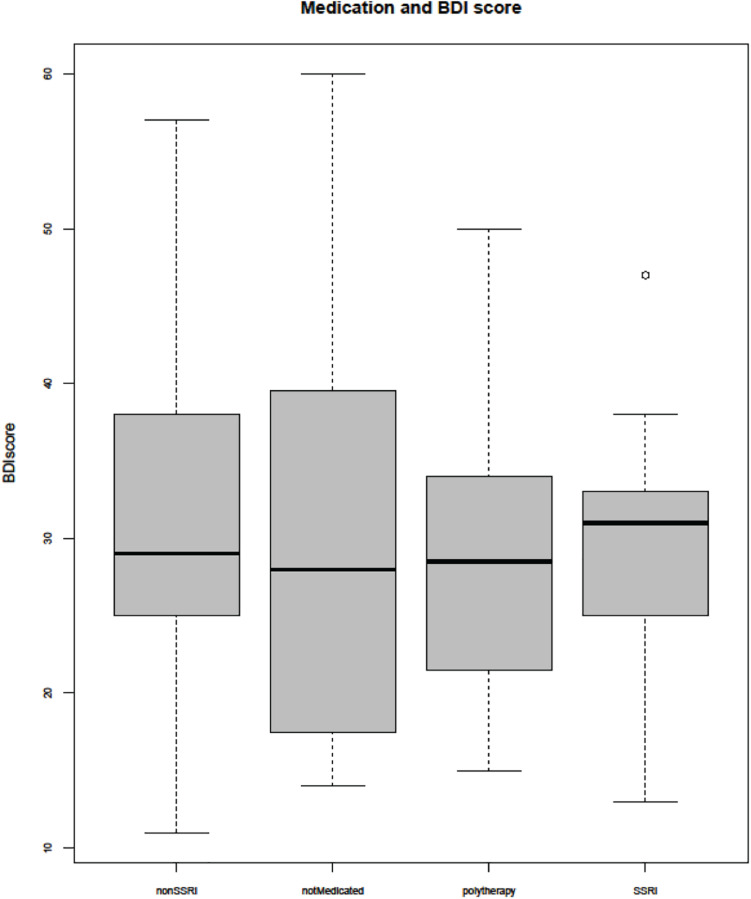
Beck Depression Inventory (BDI) score and medication. SSRI, selective serotonin reuptake inhibitor.

The non-medicated subjects did not differ in symptom severity measured by BDI from those treated with SSRI or non-SSRI (Figure 1). No correlations were observed between symptom severity (BDI score) and age or BP.

Measured by BDI, symptom severity was positively associated with SC under the stress condition (*r* = 0.293, *p* = 0.0059). A weak, but still significant, correlation was observed between the BDI score and RR in the relaxed state (*r* = 0.217, *p* = 0.043).

In the next step, ANS reactivity was assessed as the difference between baseline and stress condition, stress condition, and relaxed state, as well as baseline and relaxed state. The differences in SC levels between stress and relaxed states correlated with symptom severity measured by the BDI-II (*r* = 0.221, *p* = 0.037). The correlation between symptom severity and SC differences between baseline and stress conditions (*r* = ^–^0.195) did not reach the significance level (*p* = 0.068), nor did the other ANS reactivity measures.

Regarding medication with antidepressants, RR in the relaxed state was significantly lower in medicated subjects than in unmedicated subjects (*M* = 12.54 vs. 14.70 breaths/min, *p* < 2.2 × 10^–16^); the other parameters did not differ between the groups. Because of the small sample size, we decided not to run further analyses (between different groups of antidepressants, for example).

### Age

Higher age was related to higher SC, higher RR, and lower HRV at baseline as well as in the relaxed state. Under stress conditions, no relationship between age and electrophysiological parameters was observed (Table 3). Regarding correlations between ANS reactivity indices (differences between stages) and age, the strongest effects of age could be seen in the changes in frequency parameters of HRV (LF and HF) between baseline and stress exposure. Age was positively related only to systolic BP (*r* = 0.303, *p* = 0.0039).

**TABLE 3:** Age and electrophysiological parameters.

Parameter	SC (rho)	ST (rho)	RHR (r)	RR (r)	SDNN (rho)	RMSSD (rho)	VLF (rho)	LF (rho)	HF (rho)	LF/HF (rho)
Baseline	−0.303**	n.s.	n.s.	0.267*	−0.475***	−0.383***	−0.283**	−0.385***	−0.471***	n.s.
Stress exposure	n.s.	n.s.	n.s.	n.s.	n.s.	n.s.	n.s.	n.s.	n.s.	n.s.
Relaxed state	−0.266*	n.s.	n.s.	0.362***	−0.461***	−0.426***	−0.267*	−0.384***	−0.395**	n.s.
B-S	−0.274***	n.s.	n.s.	0.378***	−0.456***	−0.358***	−0.46***	−0.564***	−0.647***	n.s.
S-R	0.274***	n.s.	n.s.	−0.343***	0.401***	0.402***	0.281**	0.457***	0.435***	n.s.
B-R	n.s.	n.s.	n.s.	n.s.	n.s.	0.238*	−0.389***	−0.239*	−0.310**	n.s.

Statistical significance defined as *p < 2.05, **p < 2.01, and ***p < 2.001; n.s., not significant (correlation coefficient: rho, Spearman; *r*, Pearson). B-R, difference between baseline and relaxed state; B-S, difference between baseline and stress exposure; HF, high frequency; HRV, heart rate variability; LF, low frequency; RHR, resting heart rate; RMSSD, root mean square of successive differences; RR, respiration rate, SC, skin conductance; SDDN, square root of the beat-to-beat interval; SD, standard deviation; S-R, Difference between stress exposure and relaxed state; ST, skin temperature; VLF, very low frequency.

### Sex

Significant sex differences were observed in the relaxed state only: LF and the LF/HF ratio were higher in men than in women (mean 1001 vs. 2834, *p* = 0.022; mean 5.87 in men vs. 2.46 in women, *p* = 0.005; respectively).

### Body Mass Index

A higher BMI was associated with a higher ST at baseline, stress exposure, and relaxed state (*r* = 0.351, *p* = 0.0008; *r* = 0.336, *p* = 0.0014; *r* = 0.276, *p* = 0.010).

### Blood Pressure

We ran separate analyses for systolic and diastolic BP.

Systolic BP was correlated positively with RR and negatively with HRV at baseline and in the relaxed state. In the relaxed state, a negative correlation between BP and SC was observed. Under stress exposure, there was a weak negative correlation between RR and systolic BP. The correlation between systolic BP and VLF was marginally significant (*p* = 0.047) (Table 4).

**TABLE 4:** Systolic blood pressure and electrophysiological parameters.

Parameter	SC (rho)	ST (rho)	RHR (r)	RR (r)	SDNN (rho)	RMSSD (rho)	VLF (rho)	LF (rho)	HF (rho)	LF/HF (rho)
Baseline	n.s.	n.s.	n.s.	0.311**	−0.314**	n.s.	n.s.	−0.265*	−0.277*	n.s.
Stress exposure	n.s.	n.s.	n.s.	−0.237*	n.s.	n.s.	0.217*	n.s.	n.s.	n.s.
Relaxed state	n.s.	n.s.	n.s.	0.286**	−0.395***	−0.294**	−0.268*	−0.378***	−0.299***	−0.250*
B−S	−0.222*	n.s.	n.s.	0.313**	n.s.	n.s.	−0.258*	n.s.	n.s	n.s.
S−R	0.243*	n.s.	−0.226*	−0.348***	0.225*	0.249*	0.260*	n.s.	n.s.	n.s.
B−R	n.s.	n.s.	n.s.	n.s.	n.s.	0.255*	n.s.	n.s.	n.s.	n.s.

Statistical significance defined as *p < 2.05, **p < 2.01, and ***p < 2.001; n.s., not significant (correlation coefficient: rho, Spearman, *r*, Pearson). B-R, difference between baseline and relaxed state; B-S, difference between baseline and stress exposure; HF, high frequency; HRV, heart rate variability; LF, low frequency; RHR, resting heart rate; RMSSD, root mean square of successive differences; RR, respiration rate, SC, skin conductance; SDDN, square root of the beat-to-beat interval; SD, standard deviation; S-R, difference between stress exposure and relaxed state; ST, skin temperature; VLF, very low frequency.

Similarly, positive correlations between diastolic BP and RHR, as well as RR at baseline and in the relaxed state, were found. Additionally, diastolic BP was inversely associated with RMSSD in the relaxed state (Table 5). The negative correlations between diastolic BP and HRV (VLF, LF, and LF/HF) did not achieve conventional threshold levels of statistical significance (*p* = 0.074, *p* = 0.089, and *p* = 0.054).

**TABLE 5:** Diastolic blood pressure and electrophysiological parameters.

Parameter	SC (rho)	ST (rho)	RHR (r)	RR (r)	SDNN (rho)	RMSSD (rho)	VLF (rho)	LF (rho)	HF (rho)	LF/HF (rho)
Baseline	n.s.	n.s.	0.319**	0.318**	n.s.	n.s.	n.s.	n.s.	n.s.	n.s.
Stress exposure	n.s.	n.s.	n.s.	n.s.	n.s.	n.s.	n.s.	n.s.	n.s.	n.s.
Relaxed state	n.s.	n.s.	0.313**	0.220*	n.s.	−0.220*	n.s.	n.s.	n.s.	n.s.
B−S	n.s.	−0.215*	0.252*	0.252*	n.s.	n.s.	n.s.	n.s.	n.s	n.s.
S−R	n.s.	n.s.	−0.301**	−0.232*	n.s.	n.s.	n.s.	n.s.	n.s.	n.s.
B−R	n.s.	n.s.	n.s.	n.s.	n.s.	0.287**	n.s.	n.s.	−0.283**	n.s.

Statistical significance defined as *p < 2.05 and **p < 2.01; n.s., not significant (correlation coefficient: rho, Spearman, *r* = Pearson). B-R, difference between baseline and relaxed state; B-S, difference between baseline and stress exposure; HF, high frequency; HRV, heart rate variability; LF, low frequency; RHR, resting heart rate; RMSSD, root mean square of successive differences; RR, respiration rate, SC, skin conductance; SDDN, square root of the beat-to-beat interval; SD, standard deviation; S-R, Difference between stress exposure and relaxed state; ST, skin temperature; VLF, very low frequency.

No significant association was found between symptom severity and BP (systolic or diastolic).

To eliminate the impact of age or the diagnosis of arterial hypertension as confounding variables, regression analyses with both parameters and BP were conducted. After correction for the diagnosis of arterial hypertension (18 subjects), systolic BP remained a significant factor for the following parameters: RR, VLF, and SDNN at baseline; RR under stress exposure; and SC, RR, SDNN, RMSSD, and VLF in the relaxed state (only HF at baseline was no longer significant after correction for hypertension). After correction for age, only VLF in the relaxed state remained an independent predictor; for all other parameters, age was the only independent predictor of the observed correlation between electrophysiological parameters and systolic BP.

Concerning diastolic BP, after correction for age, both age and diastolic BP remained independent predictors of elevated RR at baseline, but not in the relaxed state (with age as the only independent predictor). After correction for the diagnosis of arterial hypertension, diastolic BP was no longer an independent predictor for higher RR in the relaxed state (*p* = 0.056); all other correlations remained significant.

### Tobacco Consumption (Smoking)

The only difference between smokers and non-smokers that reached statistical significance was in the mean HF under stress exposure (smokers 33.28 vs. non-smokers 39.87, *p* = 0.037).

The mean lifetime tobacco consumption measured by pack-years correlated negatively with SDNN at baseline (rho = −0.228, *p* = 0.038) and in the relaxed state (rho = −0.222, *p* = 0.046).

## Discussion

The aim of this pilot study was to compare the impact of the severity of MDD on the ANS and its reactivity measured by different parameters.

Our results suggest that changes in ANS function in patients with major depression are complex. Symptom severity, measured using the BDI-II, was associated with elevated SC under stress exposure only as well as with elevated RR in the relaxed state. Contrary to some findings in the literature, no significant correlation between symptom severity and HRV was observed.

It is noteworthy to mention that the stressor task that we used in the present study generated stimuli with subjectively perceived intensity that fell in the medium range as well as a self-perceived unpleasant (negative) valence. A distinct pattern of ANS reactivity might have been produced by a stressor task with a negatively valenced stimulus eliciting a different range of self-reported intensity (Messerotti Benvenuti et al., 2015; Hamilton and Alloy, 2016). The ANS reactivity may be modulated by both arousal and valence of the stimuli. Probing this reactivity with positively valenced material of high emotional intensity—a proxy for arousal—might not be so easily amenable to laboratory settings, however (Botzung et al., 2010; Messerotti Benvenuti et al., 2015; Staniloiu and Markowitsch, 2020). There is a lot of heterogeneity with respect to the stress tasks employed to probe ANS reactivity in depression. In our study, we used a task that recruited emotionally self-relevant imaginative and recall processes. Several functions have been reported to be impaired in patients with depression in relation to its severity, such as episodic memory, imaginative processes (self-projection), and executive functions (such as those underpinning strategic retrieval) (Williams and Kuyken, 2012; Staniloiu and Zaretsky, 2015; Schilling et al., 2021). The choice of the stress task and the purported relation between the severity of depression and extent of impairment in cognitive–affective domains described above may offer an explanatory avenue for the observed positive correlation between SC response and symptom severity. The absence or presence of correlation between symptom severity and ANS function may be also partially explained by different ranges or different assessments of the symptom severity in literature.

Regarding the methodological aspects, the following limitations should be noted. First, HRV analysis was conducted *via* photoplethysmography (pulse analysis). Some authors suggest it is more appropriate to use the term “pulse rate variability” (PRV) as a different biomarker than HRV (Yuda et al., 2020). The estimation of the fiducial point of the pulse wave (Peralta et al., 2019) was set using BioTrace software. Since HRV is still considered to be a major determinant of PRV with high correlation between both (Gil et al., 2010; Iozzia et al., 2016), we decided to use the term HRV. Second, no correction of HRV for respiration was performed in this study, which might impair the performance of the HRV analysis (Quintana et al., 2016). With the mean respiratory rates in the presented study, in average, respiratory rate was inside the HF band (0.15–0.4 Hz). If checked for every single patient and all conditions, RR was outside this range at baseline in one case (<9 breaths/min), under stress in two cases (>24 breaths/min) and in the relaxed state in 10 cases (<9 breaths/min). We conclude that in the relaxed state only, the HRV analysis might be affected by the respiratory bias.

All biomarkers used in this study were indirect measures of autonomic activity.

As expected, HRV (measured by photoplethysmography) was reduced, and the RR and RHR were elevated under stress exposure compared with the baseline or relaxed state. Only RR values differed significantly between baseline and relaxed state, either suggesting the robustness of this parameter (compared with SC, ST, or HRV with rather broad variability) or reflecting the use of slow breathing as a relaxation tool. Neither the SC nor ST values differed significantly between the three test conditions. The observed differences in age, sex, and tobacco use were consistent with those of previous studies (Dinas et al., 2013; Voss et al., 2015; Koenig et al., 2016; Takayama et al., 2019).

The positive correlation between SC response and symptom severity is contrary to the results of the literature review of Sarchiapone, with most findings showing a hypoactive electrodermal response in depressed patients (Sarchiapone et al., 2018). One possible explanation could be the choice of the stressor in the present study—recalling an unpleasant stressful experience. As some findings suggest, the attenuated SC response in depression might be stressor specific (Schwerdtfeger and Rosenkaimer, 2011). Thus, ANS arousal in patients with MDD may be different with emotional stressors than with cognitive or social stressors. In a study by Ottaviani et al. (2014), worriers showed increased SC responses during worry conditions as compared with non-worriers. For SC, the means of the absolute SC values for each period were compared. Owing to the short time of the measurement (5–1–5 min), we assume that the changes in SC should be more related to the phasic than to tonic component of SC.

On the other hand, our results indicate that age and BP do influence the ANS, especially in the relaxed state and at baseline. After regression analysis, age remained the most important influencing factor; however, for some parameters, BP remained more significant than age. Interestingly, almost no age- or BP-related changes under stress conditions could be found, which suggests impaired ANS reactivity. Our results are in agreement with previous findings of reduced HRV in subjects with elevated BP (Fonkoue et al., 2018; Grandemange et al., 2019; Dorey et al., 2020).

In our study population, the mean BP was almost at the cut off point for a diagnosis of hypertension; however, only 14 of 89 patients had been diagnosed with arterial hypertension. Hence, we suggest, in accordance with Fugger et al. (2019), that arterial hypertension seems to be underdiagnosed in patients with MDD.

The observed lower respiratory rate in medicated patients in the relaxed state was consistent with some findings pointing out the specific influence of antidepressants on respiratory activity (Warren and Solomon, 2012). Because of the small sample size, we abandoned further analyses.

## Conclusion

Symptom severity was associated with higher sympathetic arousal in an emotion-driven imaginative and retrieval-generated stressor condition and a higher respiratory rate in the relaxed condition.

Heart rate variability indices were not significantly correlated with symptom severity (measured by BDI) in any of the stages of the protocol: baseline, stress, or recovery. ANS reactivity measured from HRV index differences between the stages was not significantly correlated with the BDI score.

The present study demonstrates that the ANS changes in patients with MDD are complex, and the assessment of ANS reactivity by more parameters than HRV alone might be useful in understanding specific disease-related changes and their underlying mechanisms in depression. With respect to our results, respiratory rate can be considered a useful and easy-to-obtain marker of ANS reactivity in patients with MDD.

Furthermore, our results indicate that influencing factors, such as BP or age, can have a greater impact on ANS than symptom severity, and should, therefore, be considered in the study design and data interpretation. Elevated BP seems to be an important but possibly neglected comorbidity in patients with MDD. The relationship between BP and depression should be investigated further.

## Data Availability Statement

The raw data supporting the conclusions of this article will be made available by the authors, without undue reservation.

## Ethics Statement

The studies involving human participants were reviewed and approved by the Ehitkommission, Landesärztekammer Baden-Württemberg. The patients/participants provided their written informed consent to participate in this study.

## Author Contributions

JS, AS, and AK contributed to conception and design of the study. AK implemented the study in the clinical setting. JS collected the data, performed the statistical analysis, and wrote the first draft of the manuscript, supervised by AK. AS wrote sections of the manuscript. All authors contributed to the manuscript revision, read, and approved the submitted version.

## Conflict of Interest

The authors declare that the research was conducted in the absence of any commercial or financial relationships that could be construed as a potential conflict of interest.
